# Estimates of Medicaid and Non-Medicaid Net Prices of Top-Selling Brand-name Drugs Incorporating Best Price Rebates, 2015 to 2019

**DOI:** 10.1001/jamahealthforum.2022.5012

**Published:** 2023-01-13

**Authors:** Lisa Clemans-Cope, Marni Epstein, Jessica Banthin, Aaron S. Kesselheim, Thomas J. Hwang

**Affiliations:** 1 Health Policy Center, The Urban Institute, Washington, DC; 2 Salt Lake County Human Services Department, Salt Lake City, Utah; 3 Program on Regulation, Therapeutics, and Law (PORTAL), Division of Pharmacoepidemiology and Pharmacoeconomics, Department of Medicine, Brigham and Women’s Hospital and Harvard Medical School, Boston, Massachusetts

## Abstract

**Importance:**

The US spends far more on brand-name prescription drugs than other comparable countries. However, studies of prescription drug spending in the US are often limited because there can be substantial differences in the confidential rebates that drug manufacturers pay to Medicaid vs other payers.

**Objectives:**

To demonstrate an approach for improved estimation of Medicaid rebates through case studies of 18 top-selling drugs to better understand trends in net Medicaid and non-Medicaid spending and prices for brand-name drugs.

**Design, Settings, and Participants:**

This was a cross-sectional study of US pricing data from 2015 to 2019 derived from Medicaid State Drug Utilization data SSR Health, Medi-Span, the Federal Supply Schedule, and IQVIA. Pricing data for 18 top-selling brand-name drugs measured consistently in both SSR Health, which captures US sales reported by publicly traded companies, and IQVIA’s top US prescription drugs by nondiscounted spending in 2015 to 2019. Data were accessed and analyzed from January 2019 to June 2021.

**Main Outcomes and Measures:**

Gross and net Medicaid and non-Medicaid drug spending for the sample of 18 drugs and prices corresponding to a 30-day supply of medication.

**Results:**

Medicaid aggregate gross spending for the 18 drugs in the sample increased 173%, from $3.6 billion in 2015 to $9.9 billion in 2019, and estimated net spending after discounts increased by 119%, from $1.4 billion to $3.0 billion. Medicaid inflation-linked rebates reduced average gross price per 30-day supply by an estimated 43% in 2019, and up to 67% for individual drugs. In addition to the basic rebate, the best price provision reduced the average gross price per 30-day supply by an estimated 3% in 2019 and up to 54% for individual drugs. Between 2015 and 2019 across all study drugs, estimated average non-Medicaid net 30-day prices were between 1.9 and 2.6 times higher than Medicaid net prices. Excluding adalimumab—a spending anomaly because of the entry of a new high-cost formulation—net prices weighted by average gross spending decreased annually by 1% from 2015 through 2019 for Medicaid, while increasing by 2% for non-Medicaid payers.

**Conclusions and Relevance:**

In this cross-sectional study of 18 top-selling brand-name drugs, excluding 1 anomaly, Medicaid average net prices declined from 2015 to 2019. Simultaneously, for non-Medicaid payers, net price increased more than previously published marketwide growth rates, raising the importance of restraining drug price growth in non-Medicaid markets. Rigorous and transparent methods to estimate Medicaid discounts are imperative to understand patterns in Medicaid and non-Medicaid prices and develop policies that better align drug prices with clinical benefits.

## Key Points

**Question:**

Can a new method for estimating Medicaid discounts clarify trends in net Medicaid and non-Medicaid spending and prices for the top-selling brand-name drugs?

**Findings:**

This cross-sectional study of 18 top-selling brand-name drugs demonstrated a new method to account for the Medicaid best price discount, one component of the total Medicaid discount that is sometimes overlooked by researchers. Including the best price discount reduced the estimated Medicaid drug prices in the sample by 3% in 2019, and up to 54% for individual drugs.

**Meaning:**

The findings of this cross-sectional study suggest that rigorous and transparent methods to estimate Medicaid discounts can help reveal patterns in prices and spending, which can be helpful in developing strategies for better aligning drug prices with clinical benefits.

## Teaser

This cross-sectional study considers 18 top-selling drugs to demonstrate a new approach to improved estimation of Medicaid rebates to better understand trends in Medicaid and non-Medicaid spending and drug pricing.

## Introduction

In the US, patients and other health care payers face higher prices for brand-name prescription drugs than they would in any other comparable country.^1,2,3^ One frequent point of confusion in policy discussions regarding the need for reform in this area is estimates of net drug spending and how much they have grown. Net spending represents the amount paid by public or private payers plus the amount, if any, paid out-of-pocket by patients, after accounting for rebates and discounts from manufacturers. Although list prices are more readily available for study, net spending is critical for understanding market competition and actual payer spending on drugs. Estimates of net spending growth that average rebates across all payers may be misleading because they combine commercial, Medicare Part D, and Medicaid markets without fully adjusting for the much higher rebates and discounts received by Medicaid programs.^4,5,6^

In the US, brand-name manufacturers freely set drug list prices and then negotiate rebates with each of the different prescription drug plans in the commercial and Medicare Part D markets. By contrast, for Medicaid programs, the US Congress has set mandatory drug rebates. The basic Medicaid rebate is either 23.1% of the average manufacturer price (AMP) or the estimated “best price” (the lowest price available to wholesalers/retailers in the private sector), whichever is greater. In addition, the Medicaid inflation rebate offsets price increases in excess of inflation and is often greater than the basic rebate, particularly for drugs that have rapidly increased in price. State Medicaid programs and Medicaid managed care organizations may also negotiate confidential supplementary rebates,^7^ including through pharmacy benefit managers (PBMs), which may further decrease the overall net price that Medicaid programs pay for drugs.^8^

Given that Medicaid drug spending accounts for roughly 20% of the overall market, separating Medicaid from non-Medicaid net spending would offer more precise information to support drug pricing reform.^9^ This study responds to the need for better estimates of Medicaid and non-Medicaid net drug prices and recent research recommendations that urge more formal attempts to incorporate the best price provision into Medicaid drug price estimates.^10^ To help fill this research gap, we developed a method to more accurately estimate the Medicaid rebate, separately estimate the basic rebate (including the best price provision), and the inflation-linked rebate. We applied this method to top-selling brand-name drugs.^11^ Then, we calculated increases over a recent period in non-Medicaid net prices to inform current policy debate.

## Methods

Because this study used only deidentified secondary data and non-human research, it was not subject to review by the Urban Institute institutional review board or to informed consent. The study followed the Strengthening the Reporting of Observational Studies in Epidemiology (STROBE) reporting guidelines.

We obtained quarterly utilization data for Medicaid claims (fee-for-service and managed care) from 2015 through 2019, and drug launch dates from publicly available data files from the Centers for Medicaid & Medicare Services (CMS). We obtained wholesale acquisition cost (WAC) data from SSR Health and Medi-Span and estimates of marketwide total gross and net sales from SSR Health.

This analysis does not use CMS data sources to estimate AMP because of differences in price estimates across CMS data sources and gaps in data. For example, National Average Drug Acquisition Cost data excludes drugs that are inhaled, infused, instilled, implanted, or injected (5i drugs). We estimated AMP by adjusting WAC data from SSR Health and Medi-Span, differing from our previous research^12,13^ that estimated AMP by merging publicly available data sources published by CMS, which included National Average Drug Acquisition Cost data, State Drug Utilization Data (SDUD), and Average Sales Price data.

We used Federal Supply Schedule (FSS) prices published by the US Department of Veterans Affairs (VA) to estimate the Medicaid best price, using the FSS price available to all direct federal purchasers and the *Big Four* (the VA, US Department of Defense, the US Public Health Service [including Indian Health Service], and the US Coast Guard) price available to certain federal purchasers. We obtained inflation data from the US Bureau of Labor Statistics.

### Sample of Top-Selling Brand-name Drugs

We selected top-selling drugs based on marketwide data because product mix in Medicaid is different from that of the non-Medicaid mix. From IQVIA’s publicly available US marketwide data on the 20 top drugs by nondiscounted spending (unadjusted for discounts/rebates) in 2015 to 2019, we identified 18 drugs with definitions corresponding to SSR Health, after excluding 2 drugs (Lantus Solostar and Victoza 3-Pak) for which the included formulations differed substantially from the top-selling drugs identified in the study sample (eMethods in Supplement 1). The sample accounted for approximately 14.8% of total Medicaid drug spending in 2019.^14^ Estimates of a 30-day supply of each drug was based on the defined daily dose per product.^15^ Pembrolizumab (Keytruda), rituximab (Rituxan), and ustekinumab (Stelara) are primarily practitioner-administered medications rather than patient administered via outpatient pharmacies.

### Estimating Medicaid Manufacturer Drug Rebates

The Medicaid drug rebate is based on the AMP, which is the confidential average drug price paid to the manufacturer by the retail pharmacies and wholesalers that distribute them in the US. The AMP is not available to the public, but the Congressional Budget Office (CBO) has found it to be approximately 91% of the WAC.^16^ For this analysis, we extracted WAC at the NDC-9 level from SSR Health and Medi-Span. Practitioner-administered medications may be subject to different discounting practices than medications dispensed in retail pharmacies.^17^ Because the average sales price net of discounts and rebates in the private sector for practitioner-administered drugs generally varies by less than 5% on average from WAC,^18^ we also relied on WAC for these products.

### Estimating the Medicaid Best Price

*Best price* is the lowest price paid (including discounts) by wholesalers and retailers in the commercial sector, and is incorporated into the Medicaid basic rebate, as defined previously (either 23.1% of estimated AMP or estimated AMP minus best price [whichever is greater], with only some exceptions). Given that the best price is also confidential, we used the FSS price as a surrogate for the best price provision of the Medicaid rebate. The FSS price is based on the prices that manufacturers charge their most favored commercial customers and is, on average, lower than best price because it may include additional discounts for federal customers, such as the VA.^19^ The Federal Ceiling Price (FCP) is the maximum price that manufacturers can charge the Big Four for brand-name drugs. The Big Four price cannot exceed the FCP and is often less because it includes additional discounts provided to the Big Four agencies plus additional discounts when the nonfederal AMP rises faster than inflation.^16^ We compared the FSS price with the Big Four price and estimated FCP to determine when the FSS price was a good estimate of best price. Because FSS prices cannot generally increase faster than inflation during a contract period (typically 5 years),^20^ we smoothed the FSS prices over consecutive contract periods (details available in the eMethods of Supplement 1).

### Computing the Medicaid Basic Rebate

Finally, we applied our best price estimate to the formula for determining the Medicaid basic rebate, before computing the inflation-linked rebate. To estimate the Medicaid basic rebate provisions, we calculated the basic rebate for brand-name drugs at the NDC-9 level (across package sizes) in each quarter as the either 23.1% of estimated AMP or estimated AMP minus our best price estimate, whichever was greater, with best price estimated as the lowest smoothed FSS price among package sizes in the NDC-9 group.

To compute the additional inflation-linked Medicaid rebate, we estimated baseline AMP as 91% of the WAC from the earliest launch quarter within the NDC-9^16^ and calculated the inflation factor using the consumer price index for all urban consumers. Consistent with regulations in effect during the study period, the maximum Medicaid rebate was capped at 100% of AMP. To estimate total Medicaid rebate amounts for each drug, we computed the unit rebate from both the basic rebate and inflation-linked rebate as a percent of AMP at the NDC level and applied that to total Medicaid spending. Prices were standardized to a 30-day supply^15^ (details available in eMethods of Supplement 1).

### Estimating Net Medicaid and Non-Medicaid Spending

We present 2 main estimates: gross spending defined as total spending from all sources, including by payers and patients, before rebates and discounts; and net spending defined as spending after rebates and discounts. We used data on total gross spending by all payers at invoice prices (without pharmacy markups) published by IQVIA; Medicaid gross spending at retail prices (ie, higher than invoice prices) published by CMS; and total net spending by all payers published by SSR Health.

To estimate non-Medicaid net prices, we first used net spending and rebate estimates from SSR Health to estimate spending by all payers at WAC prices, which represent the manufacturers’ prices to wholesalers before any discounts or rebates to payers. We estimated Medicaid spending at WAC prices by applying a similar ratio of Medicaid share of gross spending to total WAC spending. Then we computed Medicaid and non-Medicaid total net spending (more details are available in the eEstimates in Supplement 1). We used these estimates to compute Medicaid and non-Medicaid net spending to total WAC spending ratios. Finally, to estimate Medicaid and non-Medicaid net 30-day prices, we applied these ratios to the 30-day WAC price from Medi-Span, using the average 30-day WAC price by product, weighting by gross Medicaid sales. Because adalimumab (Humira) accounted for a substantial share of total Medicaid gross spending and it experienced a large shift toward new formulations during the study period that were less subject to the inflation-linked rebate, we examined changes in net prices for the cohort with and without adalimumab.

### Statistical Analysis

All estimates of drug spending were expressed in nominal terms. Because a simple average of prices across drugs is subject to distortion from high-cost drugs with very low volume, we weighted estimates of Medicaid and non-Medicaid gross and net spending, prices, and rebates by average gross Medicaid spending by National Drug Code (NDC) at the NDC-11 level. The NDC number identified manufacturer, drug, and package information during the 2015 to 2019 period. The IQRs for key estimates are presented in the eEstimates in Supplement 1.

Data were accessed and analyzed from January 2019 to June 2021 using Stata software, version 16.1.

## Results

### Gross and Net Medicaid Spending

Medicaid gross spending on the 18 top-selling brand-name drugs was $3.6 billion in 2015, growing 173% to $9.9 billion in 2019 (Table 1). The growth in Medicaid gross spending for the 18 brand-name drugs was led by adalimumab at $810 million in 2015 and growing to $2.2 billion in 2019 (Table 2). The next 2 top spending drugs were bictegravir/emtricitabine/tenofovir alafenamide (Biktarvy), which first became available in 2018, and in 2019, accounted for $1.0 billion in spending, and lisdexamfetamine (Vyvanse), which in 2019 accounted for $974 million in spending. The Medicaid market share of total gross spending for these drugs was approximately 8%.

**Table 1.** Medicaid Gross and Net Spending, Prices, and Rebates for 18 Top-Selling Brand-Name Drugs, 2015 to 2019

Medicaid spending or price^a^	2015	2016	2017	2018	2019
All payer (Medicaid and non-Medicaid) retail spending, $B^b^	55.6	70.3	84.9	101.6	120.1
Medicaid aggregate gross spending, $B (market share %)^c^	3.6 (7)	5.5 (8)	7.1 (8)	8.3 (8)	9.9 (8)
WAC/30-d, $^d^	2891	3262	3567	3822	4029
Gross price/30-d, $^e^	2631	2968	3246	3478	3667
Medicaid rebate estimates and characteristics					
Basic rebate estimated as 23.1% of estimated AMP/30-d, $	608	686	750	803	847
Basic rebate estimate as 23.1% of estimated AMP or BPP/30-d, $	653	757	839	907	953
Additional rebates with BPP/30-d, $	46	71	89	104	106
No. of drugs likely to trigger BPP^f^	7	7	6	7	7
Drugs estimated to trigger BPP, No. (weighted %)^g^	67 (21)	77 (22)	78 (20)	90 (17)	71 (11)
Uncapped inflation rebate/30-d, $^h^	838	1158	1398	1447	1566
Total capped Medicaid rebate amount/30-d, $^i^	1492	1915	2237	2354	2519
Net price, 30-d, $^j^	1139	1053	1010	1125	1149
Rebate-related net price discount of gross price, 30-d, %^k^	62	68	71	70	70
Medicaid aggregate net spending, $B	1.4	1.7	2.0	2.5	3.0

Abbreviations: AMP, Average Manufacturer Price; BPP, Best Price Provision; NDC, National Drug Code; SDUD, Medicaid State Drug Utilization Data; WAC, Wholesale Acquisition Cost.

^a^ Estimated Medicaid spending and prices of 18 top-selling brand-name drugs, weighted by average gross Medicaid spending 2015 to 2019.

^b^ All-payer retail spending by product was collected from IQVIA. All spending, prices, and rebates were weighted by average quarterly gross Medicaid spending at the NDC-11 level in the 5-year period to compute annual estimates by drug. Drugs are aggregated to an annual index, weighted by average gross Medicaid spending in the 5-year period. Prices were standardized to a 30-d supply by product when the prescription exceeded 30 d.

^c^ The Medicaid market share was computed as gross Medicaid spending from SDUD divided by all-payer retail spending by product from IQVIA.

^d^ The WAC were extracted at the NDC-9 level from SSR Health and Medi-Span; study analysis of overlap data showed that WAC prices in SSR Health and Medi-Span are highly consistent.

^e^ Medicaid gross price was the estimated AMP.

^f^ The BPP was triggered by Eliquis, Enbrel, Januvia, Jardiance, Remicade, Stelara, Symbicort, and Vyvanse. Counts of the number of 18 top-selling brand-name drugs estimated to trigger the BPP and the inflation cap include the number with ≥1 NDC that triggers the provision.

^g^ Percentage of gross Medicaid spending associated with NDCs that triggered the BPP.

^h^ Only Eliquis triggered the inflationary rebate cap at 100% AMP (2017-2019) associated with NDCs related to approximately ≤4% of gross Medicaid spending.

^i^ Total capped Medicaid rebate amount was computed as the basic plus inflationary rebates, capped at the AMP.

^j^ Medicaid gross price estimated by the AMP per 30-d supply minus total Medicaid rebate amount, average per 30-d supply.

^k^ Because this estimate was weighted by average gross Medicaid spending in 2015-2019, it differs slightly from a simple computation that could be computed from the estimates in this table (Medicaid net price/gross price).

**Sources: **Medicaid State Drug Utilization Data, Federal Supply Schedule, SSR Health, and Medi-Span.

**Table 2.** Medicaid Gross Spending and Average Gross Price Savings With Discounts for 18 Top-Selling Brand-name Drugs, 2015 to 2019^a^

Drug brand name	Drug generic name(s)	Gross spending, $M		Savings with inflation rebate/30-d, $		Savings with BPP/30-d, $		Gross price/30-d, $^b^		Rebate discount, % of gross price/30-d	
		2015	2019	2015	2019	2015	2019	2015	2019	2015	2019
Biktarvy^c^	Bictegravir, emtricitabine, and tenofovir alafenamide	ND	1040.7	ND	24	ND	0	ND	2785	ND	24
Eliquis	Apixaban	31.1	366.9	66	172	56	246	332	458	60	100
Enbrel	Etanercept	446.1	555.5	1656	3297	0	1	3046	4905	77	90
Genvoya	Elvitegravir, cobicistat, emtricitabine, and tenofovir alafenamide	1.6	792.9	0	276	0	0	2346	2785	23	33
Humira	Adalimumab	810.4	2222.1	1761	3055	0	0	3218	5196	78	82
Ibrance	Palbociclib	42.8	302.3	0	1056	0	0	8968	10 773	23	33
Januvia	Sitagliptin	336.2	553.0	193	315	1	0	393	530	72	83
Jardiance	Empagliflozin	8.8	255.3	48	186	27	36	372	533	43	65
Keytruda	Pembrolizumab	1.9	257.4	0	50	0	0	2791	3074	23	25
Opdivo	Nivolumab	7.9	168.0	58	373	0	0	10 512	11 706	24	26
Remicade	Infliximab	181.6	185.2	324	463	44	25	1006	1196	60	64
Rituxan	Rituximab	116.6	145.6	806	1301	0	0	2209	2807	60	69
Stelara	Ustekinumab	85.7	474.9	4532	8426	997	2687	12 436	16 863	67	89
Symbicort	Budesonide/formoterol	456.8	609.7	101	157	52	2	256	323	83	72
Tecfidera	Dimethyl fumarate	219.7	313.7	819	2608	0	0	5128	7240	39	59
Trulicity	Dulaglutide	3.8	333.2	48	243	0	0	557	787	32	54
Vyvanse	Lisdexamfetamine dimesylate	749.7	973.6	76	133	0	5	182	250	69	80
Xarelto	Rivaroxaban	119.8	344.1	95	202	0	0	321	451	52	68
Total average		3620.5	9894.0	838	1566	46	106	2631	3667	62	70

Abbreviations: AMP, Average Manufacturer Price; BPP, Best Price Provision; ND, no data; NDC, National Drug Code.

^a^ Estimated Medicaid gross spending and average gross price savings with inflation rebates, BPP, and net price discount per 30-day supply of 18 top-selling brand-name drugs, weighted by average gross Medicaid spending.

^b^ Medicaid gross price is the estimated AMP (details in the Methods and eMethods in Supplement 1). All spending, prices, and rebates are weighted by average quarterly gross Medicaid spending at the NDC-11 level in the 5-year period to compute annual estimates by drug. Drugs are aggregated to an annual index, weighted by average gross Medicaid spending in the 5-year period. Prices were standardized to a 30-d supply by product when the prescription exceeded 30 days.

^c^ Biktarvy was launched in 2018, therefore only partial data were available.

**Sources:** Medicaid State Drug Utilization Data, Federal Supply Schedule, SSR Health, and IQVIA.

Including all of the Medicaid estimated rebates, the rebate-related net price discount was 62% of gross price in 2015 per 30-day supply, growing to 70% of gross price in 2019. After accounting for estimated discounts under all federal rebates, Medicaid net spending for this sample was $1.4 billion in 2015, growing 119% to $3.0 billion in 2019. All estimates of gross and net spending in Table 1 are weighted by Medicaid gross spending by product at the NDC-11 level during 2015 to 2019.

We found wide variation in the Medicaid estimated rebate adjustment across drugs. As a percent of gross price per 30-day supply (estimated by AMP), the estimated discounts in 2015 ranged from 23% (Genvoya, Ibrance, and Keytruda) to 83% for the combination inhaler budesonide/formoterol (Symbicort). The 2019 estimated discounts ranged from 24% (Biktarvy) to 100% for apixaban; although surprisingly, 100% discounts can occur.^21^ Rebates increased over time for all the study drugs, except for budesonide/formoterol.

### Components of Medicaid Rebate-Related Discounts

The largest Medicaid rebates for this study sample were derived from the inflation-linked rebate, which grew from an average of $838 per 30-day supply in 2015 to $1566 in 2019. This reduced the average gross price per 30-day supply by an estimated 42.7% ($1566 of $3667) overall in 2019, with a high of 67.2% ($3297 of $4905) for etanercept (Enbrel). During the study period, only the anticoagulant apixaban (Eliquis) triggered the total rebate cap of 100% of estimated AMP in 2017 to 2019.

Compared with the basic rebate of 23.1% of AMP, the best price provision increased the basic rebate by $46 per 30-day supply in 2015 (1.7% of AMP), rising to $106 per 30-day supply in 2019 (2.9% of AMP). The Medicaid best price provision applied to fewer drugs than did the inflation-linked rebate; however, the best price provision was substantial for several drugs, including apixaban (Eliquis), ustekinumab (Stelara), and empagliflozin (Jardiance).

### Non-Medicaid Net Spending

We found that the estimated average net non-Medicaid prices (Medicare Part D and commercial price) were between 1.9 and 2.6 times higher than average net Medicaid prices between 2015 and 2019 for the drugs in this study (Table 3). For each drug in each year, average net non-Medicaid prices were almost always substantially higher than Medicaid net prices. In 2019, estimated non-Medicaid net prices for apixaban, ustekinumab, etanercept, rituximab (Rituxan), lisdexamfetamine, and dimethyl fumarate (Tecfidera) were more than twice Medicaid net prices. Estimated net price decreased for 10 drugs in Medicaid between 2015 and 2019, while the same was true for 7 drugs in the non-Medicaid market. For adalimumab, the ratio of non-Medicaid to Medicaid average net 30-day price fell dramatically from 5.7 in 2018 to 1.7 in 2019 as new formulations that were not subject to the inflation-linked rebate became more prevalent than older formulations.

**Table 3.** Estimated Medicaid Average Net Prices, Ratio of Non-Medicaid to Medicaid Average Net Prices, and Average Best Price for 18 Top-Selling Brand-name Drugs, 2015 to 2019

Brand-name drug	Average net price weighted by average gross Medicaid spending for 30-d supply, $														
	Medicaid^a^^,^^b^					Non-Medicaid, at product level					Ratio of Non-Medicaid to Medicaid				
	**2015**	**2016**	**2017**	**2018**	**2019**	**2015**	**2016**	**2017**	**2018**	**2019**	**2015**	**2016**	**2017**	**2018**	**2019**
Biktarvy^c^	ND	ND	ND	2059	2069	ND	ND	ND	2415	2288	ND	ND	ND	1.2	1.1
Eliquis^d^	149	108	39	7	7	238	245	252	230	220	1.6	2.3	6.5	34.7	31.6
Enbrel	896	663	595	603	995	2799	3380	3436	3478	3713	3.1	5.1	5.8	5.8	3.7
Genvoya^e^	NA	1822	1797	1812	1793	NA	1942	2122	2184	2160	NA	1.1	1.2	1.2	1.2
Humira	753	597	541	677	2249	2879	3236	3680	3877	3830	3.8	5.4	6.8	5.7	1.7
Ibrance	7290	7530	7691	7587	7691	9822	10 171	10 850	10 077	10 509	1.3	1.4	1.4	1.3	1.4
Januvia	110	100	96	91	87	221	210	188	163	138	2.0	2.1	2.0	1.8	1.6
Jardiance	218	186	183	169	181	145	206	212	206	176	0.7	1.1	1.2	1.2	1.0
Keytruda^f^	2333	2159	2269	NA	NA	2984	3023	3136	NA	NA	1.3	1.4	1.4	NA	NA
Opdivo	8278	8514	8482	8909	9001	11 134	11 324	11 634	12 100	11 907	1.3	1.3	1.4	1.4	1.3
Remicade	337	268	213	291	280	740	803	734	589	497	2.2	3.0	3.4	2.0	1.8
Rituxan	876	837	840	886	874	2282	2310	2618	2883	2881	2.6	2.8	3.1	3.3	3.3
Stelara	4550	3841	3261	2506	2136	11 461	13 392	12 379	11 558	11 620	2.5	3.5	3.8	4.6	5.4
Symbicort	47	49	60	74	82	160	117	99	65	55	3.4	2.4	1.6	0.9	0.7
Tecfidera	3873	3349	3283	2952	3149	5331	6075	6585	6507	6864	1.4	1.8	2.0	2.2	2.2
Trulicity	503	395	381	367	363	508	409	429	423	377	1.0	1.0	1.1	1.2	1.0
Vyvanse	64	58	56	56	59	145	163	170	186	189	2.3	2.8	3.0	3.3	3.2
Xarelto	161	149	142	137	135	225	230	216	188	159	1.4	1.5	1.5	1.4	1.2
Total	1187	1135	1091	1109	1505	2513	2720	2860	2842	2864	2.1	2.4	2.6	2.6	1.9

Abbreviations: AMP, Average Manufacturer Price; NDC, National Drug Code; NA, not available; ND, no data; SDUD, Medicaid State Drug Utilization Data; WAC, Wholesale Acquisition Cost.

^a^ The computation of Medicaid net price differs from Table 1 because the data do not support the computation of net price as AMP minus rebate at the unit level for non-Medicaid prices; thus, we used the rebate percent discount applied to total spending at WAC. Net prices are estimated by estimating sales at WAC by multiplying SSR Health net sales at the product level by the product-level weighted average WAC and applying the product to the net discount published by SSR Health. Medicaid and non-Medicaid WAC sales are estimated by applying the share of the ratio of Medicaid spending from SDUD to total retail spending from IQVIA to sales at WAC. Total net spending as a share of WAC spending is calculated by dividing our estimate of total net spending, calculated as retail spending minus rebate amounts, by estimated sales at WAC for Medicaid and non-Medicaid, respectively. Medicaid rebates are estimated in this study; non-Medicaid rebates are estimated as total rebates minus Medicaid rebates, where total rebates are calculated as retail spending from IQVIA minus net spending from SSR Health. To estimate net price, we apply this percentage to the estimated WAC price for a 30-day supply, using the distribution of package sizes within NDC-11 groups from Medicaid SDUD. Best price was estimated using a smoothing algorithm for FSS prices.

^b^ Spending, prices, and rebates reported by drug are weighted by average quarterly gross Medicaid spending at the NDC-11 level in the 5-year period. Drugs are aggregated to an annual index, weighted by average gross Medicaid spending in the 5-year period. This approach captures increases in the prices of individual drugs, holding the basket of drugs and formulas constant.

^c^ Biktarvy was launched in 2018, therefore only partial data were available.

^d^ For 2019, we estimated a 100% rebate as a percentage of AMP for apixaban (Table 2), which is the generic form of Eliquis. This correlates to a net price of $7 rather than $0 (Table 3) because the net price uses a baseline of gross price, which is slightly higher than AMP.

^e^ IQVIA did not publish retail sales for Genvoya in 2015.

^f^ SSR Health did not publish net sales for Keytruda in 2018-2019.

**Sources: **Medicaid State Drug Utilization Data, Federal Supply Schedule, SSR Health, and IQVIA.

### New Formulations of Adalimumab

Overall, average Medicaid net prices for this sample of 18 top spending drugs increased by 7% from 2015 through 2019, while prices increased by 3% for non-Medicaid payers (Table 4). The higher growth rate for Medicaid was associated with a 36% jump in net prices in 2018 to 2019 that accompanied the introduction of new formulations of adalimumab. Adalimumab’s large share of total spending, approximately 22% in both years (calculation not shown), was strongly associated with the overall change in the size of the discount as a share of gross price across all drugs during the study period. New citrate-free higher concentration^22^ and autoinjector pen formulations^23^ of adalimumab approved by the US Food and Drug Administration and introduced in 2018 affected our calculations of average rebates. Although the new formulations had similar gross prices to the older products, the new formulations were not subject to the inflation-linked rebate that the older products received. The inflationary rebate was calculated at the NDC-9 level, resulting in higher net prices for the new formulations as compared with the older formulations. This produced a large price increase in the average price for adalimumab across all formulations and a subsequent large price increase in the overall sample of drugs given adalimumab’s large share of total spending. Excluding adalimumab, average Medicaid net prices fell by 1% from 2015 through 2019, but rose 2% for non-Medicaid payers during the same period.

**Table 4.** Changes in Medicaid and Non-Medicaid Net Prices for 18 Top-Selling Brand-name Drugs With and Without Adalimumab, 2015 to 2019

Study period	Total net 30-d price changes, weighted by average gross Medicaid spending^a^			
	18 Top-selling brand-name drugs, %		Adalimumab excluded, %	
	Medicaid	Non-Medicaid	Medicaid	Non-Medicaid
2015-2016	–4	8	–3	7
2016-2017	–4	5	–3	2
2017-2018	2	–1	–2	–3
2018-2019	36	1	3	2
Average annual growth rate	7	3	–1	2

Abbreviations: NDC, National Drug Code; SDUD, Medicaid State Drug Utilization Data; WAC, Wholesale Acquisition Cost.

^a^ The average net price of a 30-day supply in each year is weighted by average annual gross Medicaid spending by product over the 2015-2019 period. Underlying estimates of Medicaid and non-Medicaid prices used to compute growth rates are available in Table 3. Net prices are estimated by estimating sales at WAC by multiplying SSR Health net sales at the product level by the product-level WAC to the net discount published by SSR Health. Medicaid and non-Medicaid WAC sales are estimated by applying the share of the ratio of Medicaid spending from SDUD to total retail spending from IQVIA to sales at WAC. Total net spending as a share of WAC spending is calculated by dividing our estimate of total net spending, calculated as retail spending minus rebate amounts, by estimated sales at WAC for Medicaid and non-Medicaid, respectively. Medicaid rebates are estimated; non-Medicaid rebates are estimated as total rebates minus Medicaid rebates, where total rebates are calculated as retail spending from IQVIA minus net spending from SSR Health. To estimate net price, we applied this percentage to the estimated WAC price for a 30-day supply, using the distribution of package sizes within NDC-11 groups from Medicaid SDUD. Best price is estimated using a smoothing algorithm for Federal Supply Schedule prices.

**Sources:** Medicaid State Drug Utilization Data, Federal Supply Schedule, SSR Health, and IQVIA.

## Discussion

Demonstrating our proposed method for estimating Medicaid rebates in this cross-sectional study of 18 top-selling drugs, we developed an estimate of trends in net Medicaid and non-Medicaid spending for top-selling prescription drugs. Medicaid rebates reduced the gross drug prices by 62% to 70% in 18 blockbuster drugs between 2015 and 2019; rebates for individual drugs ranged from 23% to 100% of estimated AMP. The inflation-linked rebate accounted for a large share of the Medicaid rebate compared with the best price rebate. Best price rebates reduced the net prices of approximately one-third of the 18 brand-name drugs assessed, with an overall 2% to 3% reduction of AMP across the 18 drugs between 2015 and 2019.

After accounting for the Medicaid rebates in the data and excluding adalimumab (because of its large shifts toward new formulations during the study period), we found that net prices for Medicaid drugs decreased by 1%, whereas net prices increased approximately 2% annually for non-Medicaid drugs from 2015 through 2019. These estimates are largely consistent with previous estimates that use a price index approach.^24^ For example, a recent analysis across all US payers from 2012 to 2017 found a 12% annual increase in list prices, with net prices increasing only 3%.^4^ A 2022 CBO report found that net prices for Medicaid and Medicare Part D drugs increased approximately 7% annually between 2015 and 2018^25^ and an estimate of marketwide net price changes for brand-name, single source drugs was also 7%,^8^ somewhat higher than this study’s estimates. Our study adds to these previous estimates by raising the possibility that marketwide averages of net price growth in other studies likely mask a decline in net Medicaid prices and faster growth in Medicare Part D and commercial markets. For example, a study^4^ that reported 3% annual increases in net prices in 2012 to 2017 marketwide may mask different trends by payer.

Several important policy implications emerge from the finding that net prices generally declined in the Medicaid market but increased in the non-Medicaid market among this cohort of 18 top-selling drugs. First, Medicaid statutory rebates are effectively controlling net price growth over time but can be circumvented when new formulations are introduced.^26^ Although a 2021 CMS rule limits the ability of manufacturers to exploit this loophole,^27^ the new rule does not apply to all new formulations. The American Rescue Plan Act of 2021 ends the cap on the Medicaid rebate as of January 2024, and our estimates show that this change will likely yield additional savings for Medicaid. Second, little information is available on the net prices paid by Part D and commercial plans, which are currently at the center of various proposed drug pricing reforms, and likewise little information is available on the net prices paid by Medicaid. In this study, we found that the estimated average net non-Medicaid prices (Medicare Part D and commercial price) were 1.9 to 2.6 times higher than average net Medicaid prices between 2015 and 2019, close to the CBO estimate of 2.9 in 2017 for the ratio of net Medicare Part D prices to net Medicaid prices for brand-name drugs. This project aimed to improve the methodology for estimating those net prices, starting with data commonly used by researchers and the industry. As future policy makers consider policies to lower net drug prices in both Part D and commercial plans,^28^ understanding historical net price growth for these payers is also critical.

### Limitations

This study was limited by a sample of 18 top-selling drugs; ie, with a larger sample, incorporating the best price discount into estimates of total Medicaid discounts may differ. In addition, we could not separate rebate-adjusted net spending by payer within non-Medicaid spending (eg, Medicare Part D vs commercial). Medicaid total net spending as measured by SSR Health data includes all rebates and discounts provided by manufacturers, including 340B discounts, which are not estimated in this study. Given that the 340B program accounted for approximately 6% of the total US drug market in 2016 and increased during the study period,^29,30^ this study may overestimate Medicaid rebates, and therefore, underestimate net prices. We attributed the difference between net and gross spending as the Medicaid rebate without estimating the 340B program. This is an area for future study. Finally, computation of the inflation rebates at the NDC-9 level applies inflation rebates for the original Humira to the citrate-free formulation, which would overestimate the Medicaid rebate; this limitation would apply to any other drug with a formulation that was able to exploit this loophole. Additional limitations are described in the eLimitations in Supplement 1.

## Conclusions

This cross-sectional study suggests that marketwide averages of growth in net drug prices may mask important differences among payers. One key difference with respect to Medicaid is that the program generally experienced net drug price declines in recent years among a cohort of 18 top-selling drugs. Conversely, other payers, ie, Medicare Part D and commercial plans, likely experienced faster net price growth than the marketwide averages. A major implication is that studies finding evidence of lower rates of overall price growth are in part associated with government policies explicitly designed to control Medicaid price growth. This study showed that proposals to slow drug price growth have a much larger task than commonly assumed in restraining drug price growth in the commercial and Medicare Part D markets. Rigorous and transparent methods for estimating Medicaid discounts are imperative to understanding patterns in prices and spending for developing strategies to better align drug prices with clinical benefits.

## Notes

Supplement 1.
eMethods.

eEstimates.

eLimitations.

Supplement 2.
Data Sharing Statement
