# Supplementary material for: Estimates of Medicaid and Non-Medicaid Net Prices of Top-Selling Brand-name Drugs Incorporating Best Price Rebates, 2015 to 2019
Source: JAMA Health Forum. 2023 Jan 13;4(1):e225012. doi: 10.1001/jamahealthforum.2022.5012 (PMC9857139; doi:10.1001/jamahealthforum.2022.5012)
Supplement: Supplement 1. — eMethods. eEstimates. eLimitations. [file jamahealthforum-e225012-s001.pdf]

Supplemental Online Content

Clemans-Cope L, Epstein M, Banthin J, Kesselheim AS, Hwang TJ. Estimates of Medicaid and non-Medicaid net prices of top-selling brand-name drugs incorporating best price rebates, 2015 to 2019. *JAMA Health Forum.* 2023;4(1):e225012. doi:10.1001/jamahealthforum.2022.5012

eMethods.

eEstimates.

eLimitations.

This supplemental material has been provided by the authors to give readers additional information about their work.

© 2023 Clemans-Cope L et al. *JAMA Health Forum.*

eMethods

Sample of Selected Top Selling Brand-name Drugs

Brand name	Generic name(s)
Biktarvy	bictegravir, emtricitabine and tenofovir alafenamide
Eliquis	apixaban
Enbrel	etanercept
Genvoya	elvitegravir, cobicistat, emtricitabine, and tenofovir alafenamide
Humira	adalimumab
Ibrance	palbociclib
Januvia	sitagliptin
Jardiance	empagliflozin
Keytruda	pembrolizumab
Opdivo	nivolumab
Remicade	infliximab
Rituxan	rituximab
Stelara	ustekinumab
Symbicort	budesonide/ formoterol
Tecfidera	dimethyl fumerate
Trulicity	dulaglutide
Vyvanse	lisdexamfetamine dimesylate
Xarelto	rivaroxaban

© 2023 Clemans-Cope L et al. *JAMA Health Forum.*

Price Measures, Descriptions, Data Sources and Availability

For clarity, we present a table of price measures and definitions, drawing on and updating a similar table published in a CBO report.[1]

Price	Description	Details	Data Sources and Availability
Wholesale Acquisition Cost (WAC)	Manufacturer's list price to wholesalers	This is the published list price, though in practice the WAC is not the wholesaler's price; WAC is thought to be slightly higher than what retail pharmacies pay.[2]	SSR Health, Medi-Span data. Both can be purchased.
Average Manufacturer Price (AMP)	Average price that retail purchasers pay manufacturers	This is the price before rebates or discounts; a 2021 report found that AMP was 91% of WAC and 98% of the Medicaid retail price.[3]	Estimated from SSR Health and Medi-Span.
Non-federal Average Manufacturer Price (non-FAMP)	Average price that retail and non-retail nonfederal purchasers pay manufacturers	This price includes discounts; a 2021 report found that non-FAMP was 90% of AMP and 82% of WAC.[3]	Estimated as 82% of WAC from SSR Health.
Best Price (BP)	Lowest available price to wholesalers or retailers in the private sector	This price excludes government programs like the 340B drug pricing program and the VA; however, BP is used in computing the Medicaid rebate.[4] Note that BP could potentially be lower than Federal Supply Schedule (FSS) because the set of purchasers used for FSS could exclude the BP purchaser.	Not publicly available. In this study, we estimate BP using a method based on FSS prices as explained in appendix.
Federal Supply Schedule (FSS) Price	Price negotiated by the Veterans Affairs (VA) and available to government agencies other than the Big Four (i.e. not the VA, the Department of Defense, the Public Health Service including Indian Health Service and the Coast Guard).	This price is determined by statutory rules and negotiation by the VA with manufacturers; it cannot increase faster than inflation or the net price charged to the most-favored customer during contract period.[3] Often based on Most Favored Customer (MFC) price. Usually higher than the Big Four Price, although a single FSS price can be offered to the Big Four and other government agencies.	Publicly available in the FSS data file.
Big Four Price	Price negotiated by the VA and available to the Big Four (the	The Big Four price is the minimum of the FSS price and the FCP, minus any additional price	Publicly available in the FSS data file.

© 2023 Clemans-Cope L et al. *JAMA Health Forum.*

	VA, the Department of Defense, the Public Health Service including Indian Health Service and the Coast Guard).	concessions,[3] and must be no higher than the FCP or the FSS Max Cap.	
Most Favored Customer (MFC) Price	The lowest price paid by a private purchaser, including discounts.	Used in negotiations with VA to establish the FSS price, MFC is a private market price that represents purchasers on similar terms and conditions getting the best deals. Conceptually, the MFC is to the VA what Best Price is to Medicaid.	Not publicly available; it is used conceptually in this study.
Federal Ceiling Price (FCP)	Upper Limit on Price Charged to Big Four Agencies.	In the first year of contract period, the FCP is 24% off of (i.e. it is 76% of) the prior year's non-FAMP.[1]	Estimated as 76% of 82% of the previous year's WAC.
FSS Max Cap	FSS price offered to other government agencies sets the FSS Max Cap.[5]	The FSS Max Cap caps the FCP in the second and subsequent years of a contract.	We do not estimate this measure; it is used conceptually.

© 2023 Clemans-Cope L et al. *JAMA Health Forum.*

Medicaid's Rebate Calculations for Brand-Name Drugs

Basic rebate = larger of:
(1) 23.1% AMP or
(2) AMP – Best Price

+

Inflation rebate = costs in excess of baseline AMP from launch date inflated by the CPI

=

Total rebate = basic rebate + inflation rebate, capped at 100% AMP

Sample of Selected Top Selling Brand-name Drugs

The selected sample drugs were associated with 68 NDC-9 codes, combined across package sizes, in the 2015-2019 Medicaid utilization data.

Estimating Medicaid Manufacturer Drug Rebates

Estimating Medicaid's Best Price Provision

As part of this process, we compared the FSS price to the FCP and Big Four Price to determine when the FSS price was a good estimate of best price for a large sample of 156 products (including the 18 drugs analyzed in the study). Separate FSS and Big Four prices were listed for the majority (58%, weighted by Medicaid spending) of NDC-quarters, implying that the FSS price was a good estimate of best price because it was not capped by the FCP as is the Big Four price. For a share (19%) of NDC-quarters where the published FSS price was equal to the Big Four price, three-quarters of the records listed an FSS price that was the same as or higher than the FSS price from a previous period before the two prices were equal. This situation implies that the Big Four price increased (instead of the FSS price decreasing) to the FCP; for these cases we also assume that FSS prices were a good estimate of best price. For the remainder (23%) of NDC-quarters, there is no published Big Four price, so we used the FSS price to estimate best price even though the FSS price may be based on the FCP.

For the 18 products in this study, we found that single pricing was uncommon, and have thus assumed that the published FSS price reflects the most favored commercial customer discount rather than the FCP. However, single pricing was more common in the broader sample of drugs. If single pricing is more typical, this methodology may underestimate the best price and therefore overestimating the best price rebate.

Computing the Medicaid Basic Rebate including the Best Price Provision and the Inflation-linked Rebate

To estimate Medicaid's basic rebate provisions, we computed the Medicaid rebate at the NDC-9 level, combining across package sizes. We estimated AMP as the average AMP within the NDC-9 group, weighting by gross Medicaid sales. To estimate best price, we first smooth the FSS prices at the NDC-11 level to account for discontinuities and price jumps resulting from five-year contract periods. We then aggregate across package sizes to the NDC-9 level, using the lowest smoothed FSS price among package sizes for the NDC-9 group. We then choose the larger of the published FSS price or the smoothed price per NDC-9. We are assuming an increase in the FSS price from one contract to another reflects a real price increase in the price offered to the most favored customer.

We used the earliest launch quarter in the NDC-9 group (across package sizes), identifying launch quarter by NDC-11 from the Medicaid Drug Rebate Program data. We estimated baseline AMP as 91% of the WAC

© 2023 Clemans-Cope L et al. *JAMA Health Forum.*

from the earliest launch quarter within the NDC-9, using WAC prices listed in SSR Health or Medi-Span data to fill data gaps, which accounted for about half of NDCs. Though SSR Health compute the Medicaid rebate at the product-strength level, we compute it at the NDC-9 level, consistent with CMS and CBO.

To estimate total Medicaid rebate amounts for each drug, we computed the unit rebate from both the basic rebate and inflation-linked rebate as a percent of AMP at the NDC level and applied that to total Medicaid spending. To estimate the total Medicaid rebate amount, we applied the unit rebate amount as a percent of AMP at the NDC-9 level to total Medicaid spending rather than multiplying the unit rebate amount by the number of units reimbursed by Medicaid, as published in SDUD. We do this due to data quality concerns in Medicaid SDUD unit reporting.

Estimating 30-Day Supply Prices

As described in the main methods section, prices were standardized to a 30-day supply used the defined a typical daily dose per product, published by the World Health Organization,[6] following previous research.[7] For 4 products that did not have published defined daily doses (Ibrance, Keytruda, Opdivo, rituximab [Rituxan]), we determined the recommended 30-day dose based on general product guidelines. We used the following recommended 30-day doses for products without a published defined daily dose: Ibranace 30-day supply = 21 units, Keytruda 30-day supply = 71mg, based on the recommended 100 mg per 6 weeks, Opdivo 30-day supply = 480 mg, and Rituxan 30-day supply = 330 mg, based on the recommended 2g per 6 months.

Estimating Net Medicaid and Non-Medicaid Spending and Prices for Selected Top Selling Brand-name Drugs

We used net spending and rebate data from SSR Health to estimate spending at WAC prices. As a proxy for Medicaid spending at WAC as a share of total spending at WAC, we estimated a similar ratio, Medicaid's share of gross spending, by dividing Medicaid gross spending from CMS by total gross sales from IQVIA, which is 12.7%, weighted by Medicaid gross spending at the NDC-11 level. We applied this ratio to total spending at WAC to approximate Medicaid and non-Medicaid spending at WAC. As CBO estimates that sales at AMP are equal to 98% of sales at Medicaid retail prices, dividing CMS' Medicaid sales by IQVIA sales is likely to be a good estimate of Medicaid market share.

We then computed Medicaid total net spending (Medicaid gross spending from CMS minus estimated Medicaid rebates) and non-Medicaid total net spending (non-Medicaid total gross sales minus non-Medicaid rebates). Non-Medicaid gross sales was computed as IQVIA total gross sales minus Medicaid total sales from CMS and the non-Medicaid rebate was computed as IQVIA gross sales minus SSR net sales minus total estimated Medicaid rebates. We used these to compute Medicaid and non-Medicaid net spending to total WAC spending ratios. Finally, to estimate Medicaid and non-Medicaid net 30-day price, we applied these ratios to the 30-day WAC price from Medi-Span, using the average 30-day WAC price by product, weighting by gross Medicaid sales.

The table below describes the sources or calculations used to derive Medicaid and non-Medicaid prices.

© 2023 Clemans-Cope L et al. *JAMA Health Forum.*

Sources and Calculations Used to Compute Medicaid and Non-Medicaid Prices

Data point		Source or calculation
Gross spending		
**1**	Total gross spending	IQVIA
**2**	Medicaid gross spending	CMS
**3**	Non-Medicaid gross spending	= total gross spending (1) - Medicaid gross spending (2)
Rebates		
**4**	Total net spending	SSR
**5**	Total rebate percent	SSR
**6**	Medicaid rebates	Estimated, as described in methods
**7**	Non-Medicaid rebates	= total gross spending (1) - total net spending (4) - Medicaid rebates (6)
WAC spending		
**8**	Total WAC spending	= total net spending (4) * (1/total rebate percent (5))
**9**	Medicaid's share of gross spending	= Medicaid gross spending (2) / Total gross spending (1)
**10**	Medicaid WAC spending	= total WAC spending (8) * Medicaid's share of gross spending (9)
**11**	Non-Medicaid WAC spending	= Total WAC spending (8) - Medicaid WAC spending (10)
Net spending		
**12**	Medicaid net spending	= Medicaid gross spending (2) - Medicaid rebates (6)
**13**	Non-Medicaid net spending	= Non-Medicaid gross spending (3) - Non-Medicaid rebates (7)
Prices		
**14**	Medicaid net to WAC spending ratio	= Medicaid net spending (12) / Medicaid WAC spending (10)
**15**	Non-Medicaid net to WAC spending ratio	= Non-Medicaid net spending (13) / Non-Medicaid WAC spending (11)
**16**	**Medicaid price**	= Medicaid net to WAC spending ratio (14) * WAC price
**17**	**Non-Medicaid price**	= Non-Medicaid net to WAC spending ratio (15) * WAC price

Note: All calculations are by product

© 2023 Clemans-Cope L et al. *JAMA Health Forum.*

Additional Methods on the Components of Medicaid Rebate-Related Discounts

The best price provision was substantial for certain drugs. For example, for apixaban (with gross spending of $366.9 million in 2019), the Medicaid savings due to the best price provision was $246 per 30-day supply in 2019, equal to 54% of the gross price for a 30-day supply on top of the basic rebate of 23%. For ustekinumab (with gross spending of $474.9 million in 2019), the Medicaid savings due to the best price provision was $2,687 per 30-day supply in 2019, or 16% of the gross price for a 30-day supply on top of the basic rebate of 23%. Of the 18 brand-name drugs assessed, 6 to 7 were reduced by the best price provision in each year and between 11% and 22% of gross Medicaid spending was associated with drugs that triggered the best price provision.

© 2023 Clemans-Cope L et al. *JAMA Health Forum.*

eEstimates

25th percentile, 50th percentile, 75th percentile for key estimates

eTable 1. Mean, 25th percentile, 50th percentile, 75th percentile for estimated Medicaid gross and net spending, prices, and rebates for 18 top selling brand-name drugs, weighted by average gross Medicaid spending 2015-2019

Estimated Medicaid spending and prices of 18 top selling brand-name drugs, weighted by average gross Medicaid spending 2015 to 2019	Mean (25th percentile, 50th percentile, 75th percentile)				
	2015	2016	2017	2018	2019
Medicaid wholesale acquisition cost (WAC), 30-day supply, *$(25th, 50th, 75th percentile)* [3]	2,891 (353, 2,578, 3,537)	3,262 (387, 2,578, 4,335)	3,567 (422, 2,740, 4,868)	3,822 (474, 2,946, 5,370)	4,029 (503, 3,061, 5,710)
Medicaid gross price, 30-day supply, *$(25th, 50th, 75th percentile)* [4]	2,631 (321, 2,346, 3,218)	2,968 (352, 2,346, 3,945)	3,246 (384, 2,493, 4,430)	3,478 (432, 2,681, 4,887)	3,667 (458, 2,785, 5,196)
Medicaid uncapped inflationary rebate, 30-day supply, $(25th, 50th, 75th percentile)[7]	838 (76, 193, 1,761)	1,158 (97, 251, 2,472)	1,398 (126, 370, 2,926)	1,447 (143, 293, 2,922)	1,566 (157, 315, 3,055)
Total capped Medicaid rebate amount, 30day supply, $(25th, 50th, 75th percentile)[8]	1,492 (213, 600, 2,504)	1,915 (224, 767, 3,383)	2,237 (239, 912, 3,950)	2,354 (326, 834, 4,051)	2,519 (345, 919, 4,255)
Medicaid net price, 30-day supply, $(25th, 50th, 75th percentile)[9]	1,139 (109, 687, 893)	1,053 (100, 540, 870)	1,010 (100, 458, 856)	1,125 (95, 836, 1,846)	1,149 (93, 858, 1,854)

Sources: Medicaid State Drug Utilization Data (SDUD), Federal Supply Schedule Data, SSR Health, Medi-Span.
Notes: Wholesale Acquisition Cost (WAC); Average Wholesale Price (AWP); National Drug Code (NDC). All spending, prices and rebates are weighted by average quarterly gross Medicaid spending at the NDC11 level in the 5-year period to compute annual estimates by drug. Drugs are aggregated to an annual index, weighting by average gross Medicaid spending in the 5-year period. Prices were standardized to a 30-day supply by product in cases in which the prescription exceeded 30 days. See text for more details on methods.
3 We extracted WAC at the NDC-9 level from SSR Health and Medi-Span; author's analysis of overlap data showed WAC prices in SSR Heath and Medi-span are highly consistent.
4 Medicaid gross price is estimated AMP. See text for more details on methods. 5
The best price provision was triggered by Eliquis, Enbrel, Januvia, Jardiance, Remicade, Stelara, Symbicort, and Vyvanse over the study period. Counts of the number of 18 top selling brand-name drugs estimated to trigger the best price provisions and the inflation cap counts the number with at least one NDC that triggers the provision.

© 2023 Clemans-Cope L et al. *JAMA Health Forum.*

7 Only Eliquis triggered the inflationary rebate cap at 100% AMP (in 2017-2019), associated with NDCs related to about 4% or less of gross Medicaid spending.
8 Total capped Medicaid rebate amount is computed as the basic plus inflationary rebates, capped at AMP.
9 Medicaid gross price estimated by AMP per 30-day supply minus total Medicaid rebate amount, average per 30-day supply. [10] Since this estimate is weighted by average gross Medicaid spending 2015-2019, it differs slightly from a simple computation that could be computed from the estimates in this table (1 - Medicaid net price/Medicaid gross price).

eTable 2. Mean, 25th percentile, 50th percentile, 75th percentile for estimated Medicaid gross spending and Medicaid average gross price, savings due to best price and inflationary rebates, and net price discount per 30-day supply, 18 top selling brand-name drugs, weighted by average gross Medicaid spending 2015-2019

		Medicaid average rebate discount, % of gross price per 30-day supply (25th percentile, 50th percentile, 75th percentile)	
Brand name	Generic name(s)	2015	2019
**Total Average**		**62% (52%, 69%, 78%)**	**70% (64%, 80%, 82%)**
Biktarvy	bictegravir, emtricitabine and tenofovir alafenamide	n.d.	24% (23%, 24%, 24%)
Eliquis	apixaban	60% (57%, 59%, 62%)	100% (100%, 100%, 100%)
Enbrel	etanercept	77% (76%, 78%, 81%)	90% (92%, 93%, 93%)
Genvoya	elvitegravir, cobicistat, emtricitabine, and tenofovir alafenamide	23% (23%, 23%, 23%)	33% (32%, 33%, 34%)
Humira	adalimumab	78% (77%, 78%, 80%)	82% (92%, 92%, 93%)
Ibrance	palbociclib	23% (23%, 23%, 23%)	33% (32%, 33%, 33%)
Januvia	sitagliptin	72% (71%, 72%, 73%)	83% (82%, 82%, 83%)
Jardiance	empagliflozin	43% (38%, 44%, 47%)	65% (64%, 65%, 65%)
Keytruda	pembrolizumab	23% (23%, 23%, 23%)	25% (25%, 25%, 25%)
Opdivo	nivolumab	24% (23%, 24%, 24%)	26% (24%, 27%, 27%)
Remicade	infliximab	60% (57%, 60%, 62%)	64% (62%, 62%, 66%)
Rituxan	rituximab	60% (59%, 60%, 60%)	69% (69%, 69%, 70%)
Stelara	ustekinumab	67% (62%, 67%, 72%)	89% (88%, 89%, 90%)
Symbicort	budesonide/ formoterol	83% (81%, 83%, 86%)	72% (72%, 72%, 72%)
Tecfidera	dimethyl fumerate	39% (37%, 39%, 41%)	59% (59%, 59%, 60%)
Trulicity	dulaglutide	32% (31%, 32%, 32%)	54% (54%, 54%, 54%)
Vyvanse	lisdexamfetamine dimesylate	69% (69%, 70%, 71%)	80% (81%, 81%, 82%)
Xarelto	rivaroxaban	52% (51%, 53%, 54%)	68% (68%, 68%, 69%)

Sources: Medicaid State Drug Utilization Data (SDUD), Federal Supply Schedule Data, SSR Health, IQVIA total spending.tes: All spending, prices and rebates are weighted by average quarterly gross Medicaid spending at the NDC-11 level in the 5-year period to compute annual estimates by drug. Drugs are aggregated to an annual index, weighting by average gross Medicaid spending in the 5-year period. Prices were standardized to a 30-day supply by product in cases in which the prescription exceeded 30 days. See text for more details on methods. n.d. = no data. Biktarvy was launched in 2018.

© 2023 Clemans-Cope L et al. *JAMA Health Forum.*

eLimitations

The estimated non-Medicaid market price does not necessarily reflect exact Medicare Part D and commercial prices because the SSR health data include the effect of discount cards, coupons, the 340B program, VA and Department of Defense purchasers with access to statutory discounts. These limitations may explain the two cases in which we estimated that the non-Medicaid net price was below the Medicaid net price (empagliflozin and budesonide/formoterol in some years). An additional data limitation that should be explored in future research is that SSR-based estimates of non-Medicaid net prices that do not account for Medicaid rebates as we have in this study are generally far below our estimated best prices. Particularly in the later years, these non-Medicaid net prices may not always reflect pricing dynamics in the private market by Part D and commercial plans for the study drugs. Related to this point, we find that from 2017 to 2019 Medicaid net prices increase more quickly than non-Medicaid net prices (after dropping Humira) even though most of the non-Medicaid market does not enjoy Medicaid's inflation rebate protections. SSR Health estimates prices using the assumption that AMP is 3% lower than WAC, rather than 9%, which is used in this analysis (and CBO research[8]), thus estimates were difficult to compare. In addition, there may be variation in the relationship between AMP and WAC that may affect analysis of price trends over time as drugs differ in composition over years, although CBO finds that the relationship is fairly consistent across subgroups (such as specialty, nonspecialty, and high-priced drugs). For the 18 products in this study, we found that single pricing appears to be relatively uncommon, and have thus assumed that the published FSS price reflects the most favored commercial customer discount rather than the FCP. However, if the FSS actually reflects FCP, we may be estimating too low of a best price and therefore overestimating the best price rebate relative to AMP. We may also be underestimating the best price rebate if some rebates are not reflected in the FSS price. In addition, spending on physician administered drugs may not be captured entirely in these data to the extent that they are paid through medical claims rather than pharmacy claims. Physician administered drugs may also be subject to a different AMP calculation, which can include discounts, rebates, and payments in addition to sales. For such drugs, our AMP estimate based on WAC price is likely to be an overestimate, which may result in an overestimate of the rebate. Finally, the sample of 18 top-selling drugs studied here accounted for only 8% of Medicaid spending, potentially limiting the generalizability of results.

References

1. Congressional Budget Office. Prices for Brand-Name Drugs Under Selected Federal Programs. *A CBO Paper*. Published online June 2005:24.

2. Seeley E. The Impact of Pharmaceutical Wholesalers on U.S. Drug Spending. The Commonwealth Fund. doi:10.26099/6qtd-k783

3. Congressional Budget Office. A Comparison of Brand-Name Drug Prices Among Selected Federal Programs. Published February 18, 2021. Accessed April 26, 2021. https://www.cbo.gov/publication/56978

4. Dolan R, Tian M. Pricing and Payment for Medicaid Prescription Drugs. The Henry J. Kaiser Family Foundation. Published January 23, 2020. https://www.kff.org/medicaid/issue-brief/pricing-and-payment-for-medicaid-prescription-drugs/

© 2023 Clemans-Cope L et al. *JAMA Health Forum.*

5. Anderson M, Ruscus S, Lewis M. Single versus Dual Pricing and the Interplay of FCP and FSS Pricing. Presented at: May 24, 2017; American Conference Institute's 11TH "Big Four" Pharmaceutical Pricing Boot Camp. https://www.americanconference.com/11th-big-four-pharmaceutical-pricing-boot-camp/wp-content/uploads/sites/1752/2017/05/Day2_9.15_Anderson.M.Ruscus.S.Single-vs-Dual.pdf

6. World Health Organization. The ATC/DD Toolkit: Defined Daily Dose (DDD). Published 2021. Accessed September 7, 2021. https://www.who.int/tools/atc-ddd-toolkit/about-ddd

7. Anderson-Cook A, Maeda J, Nelson L. Prices for and Spending on Specialty Drugs in Medicare Part D and Medicaid: An In-Depth Analysis. Congressional Budget Office. Published March 18, 2019. https://www.cbo.gov/publication/54964

8. Congressional Budget Office. A Comparison of Brand-Name Drug Prices Among Selected Federal Programs. Published February 18, 2021. https://www.cbo.gov/system/files/2021-02/56978-Drug-Prices.pdf

© 2023 Clemans-Cope L et al. *JAMA Health Forum.*
