# Supplementary material for: Estimates of Medicaid and Non-Medicaid Net Prices of Top-Selling Brand-name Drugs Incorporating Best Price Rebates, 2015 to 2019
Source: JAMA Health Forum. 2023 Jan 13;4(1):e225012. doi: 10.1001/jamahealthforum.2022.5012 (PMC9857139; doi:10.1001/jamahealthforum.2022.5012)
Supplement: Supplement 2. — Data Sharing Statement [file jamahealthforum-e225012-s002.pdf]

Data Sharing Statement

Clemans-Cope. Estimates of Medicaid and Non-Medicaid Net Prices of Top-Selling Brand-name Drugs Incorporating Best Price Rebates, 2015 to 2019. *JAMA Health Forum*. Published January 13, 2023. doi:10.1001/jamahealthforum.2022.5012

Data
Data available: No

Additional Information
Explanation for why data not available: The main data set is SSR Health, which is not a public dataset.
